# Cardiovascular disease in people born to unmarried mothers in two historical periods: The Helsinki Birth Cohort Study 1934–1944

**DOI:** 10.1177/14034948211019792

**Published:** 2021-05-31

**Authors:** H. Maiju Mikkonen, Minna K. Salonen, Antti Häkkinen, Clive Osmond, Johan G. Eriksson, Eero Kajantie

**Affiliations:** 1 Finnish Institute for Health and Welfare, Finland; 2 Faculty of Medicine, University of Helsinki, Finland; 3 Economic and Social History, University of Helsinki, Finland; 4 Folkhälsan Research Centre, Finland; 5 MRC Lifecourse Epidemiology Unit, University of Southampton, UK; 6 Department of General Practice and Primary Health Care, University of Helsinki, Finland; 7 Singapore Institute for Clinical Sciences, Agency for Science, Technology, and Research, Singapore; 8 Department of Obstetrics and Gynaecology, and Human Potential Translational Research Programme, Yong Loo Lin School of Medicine, National University of Singapore, Singapore; 9 Children’s Hospital, Helsinki University Central Hospital and University of Helsinki, Finland; 10 PEDEGO Research Unit, MRC Oulu, Oulu University Hospital and University of Oulu, Finland; 11 Department of Clinical and Molecular Medicine, Norwegian University of Science and Technology, Norway

**Keywords:** Finland, cardiovascular disease, childhood, coronary heart disease, life course, socio-economic, stroke, unmarried mother

## Abstract

*Aims:*Socio-economic conditions in early life are important contributors to cardiovascular disease – the leading cause of mortality globally – in later life. We studied coronary heart disease (CHD) and stroke in adulthood among people born out of wedlock in two historical periods: before and during World War II in Finland. *Methods:* We compared offspring born out of wedlock before (1934–1939) and during (1940–1944) World War II with the offspring of married mothers in the Helsinki Birth Cohort Study. The war affected the position of unmarried mothers in society. We followed the study subjects from 1971 to 2014 and identified deaths and hospital admissions from CHD and stroke. Data were analysed using a Cox regression, adjusting for other childhood and adulthood socio-economic circumstances. *Results:* The rate of out-of-wedlock births was 240/4052 (5.9%) before World War II and 397/9197 (4.3%) during World War II. Among those born before World War II, out-of-wedlock birth was associated with an increased risk of stroke (hazard ratio (HR)=1.44; 95% confidence interval (CI) 1.00–2.07) and CHD (HR=1.37; 95% CI 1.02–1.86). Among those born out of wedlock during World War II, the risks of stroke (HR=0.89; 95% CI 0.58–1.36) and CHD (HR=0.70; 95% CI 0.48=1.03) were similar to those observed for the offspring of married mothers. The *p*-values for interaction of unmarried×World War II were (*p*=0.015) for stroke and (*p*=0.003) for CHD. ***Conclusions:* In a society in which marriage is normative, being born out of wedlock is an important predictor of lifelong health disadvantage. However, this may change rapidly when societal circumstances change, such as during a war.**

## Introduction

Globally, cardiovascular disease (CVD) is the leading cause of death and years of life lost; close to 18 million people die of CVD every year [1,2]. Risk factors for CVD include lower socio-economic position (SEP) throughout the life course [3–5].

In life-course epidemiology, childhood SEP is most commonly indicated by parental occupation and educational attainment [3]. These indicators, however, may not capture all aspects of inequality. One important group whose deprivation may be overlooked by these indicators is children born out of wedlock, that is, to an unmarried mother [6–9]. The few studies that have followed up these children show that they seem to carry this disadvantage throughout their lives [7,8,10,11]. At least two previous studies using Nordic data from the early part of the twentieth century have reported higher CVD risk in adult life for children from a single-parent background [8,11].

However, due to the small number of studies, the role of the specific sociohistorical context in interpreting the association between a single-parent background and CVD in later life remains unclear. It is likely that rapid changes in society, such as war, may affect the context of single-parent background by changing social norms and socio-economic resources.

Particularly in societies where marriage is the parental norm, mothers and their out-of-wedlock children form a non-standard minority that is exposed to social inequality or stigma [7,8]. In such circumstances, out-of-wedlock birth can have a detrimental impact on the child’s health throughout the life course through biological, psychosocial and/or behavioural pathways. First, socio-economic adversity can affect adult health through environmental exposures, such as malnutrition or stress during critical periods of growth and development during gestation and childhood. Second, being born or growing up in a non-standard family can cause stress and have negative effects on parent–child interaction, well-being and social relationships. Finally, a stigma associated with out-of-wedlock birth could narrow professional prospects and have effects on health in later life [4,5,12–14].

### Aims

Using the Helsinki Birth Cohort Study (HBCS) data across the life course, we investigated the associations between out-of-wedlock birth and coronary heart disease (CHD) and stroke in adulthood. The study participants were born in Helsinki, Finland, from 1934 to 1944 in a society where marriage was normative. Because the studied period includes World War II, we investigated whether the association between out-of-wedlock birth and CHD and stroke differed between those born before and those born during the war. As a secondary analysis, we performed separate analyses for men and women.

### Historical context

In Helsinki, 9% of babies were born out of wedlock from 1934 to 1944 [15]. The nuclear family predominated, and families headed by an unmarried mother were non-standard and formed a disadvantaged, potentially stigmatised group in several ways [7,8]. The infant mortality rate among the infants born out of wedlock was 75/1000 live births compared to 46/1000 live births among babies born to married mothers, and this increased during the war (118 vs. 52 deaths per 1000 live births) [15]. Moreover, children born out of wedlock were overrepresented among child welfare clients and those receiving economic assistance or being placed in out-of-home care [15].

Maternal and child health care developed rapidly during the interwar period [16]. In Helsinki, 97% of all births took place in hospitals in the late 1930s [17]. Attendance at maternal and child welfare clinics was voluntary and free of charge [16,18]. The first maternity grants were provided in 1938 for low-income mothers.

Almost 70% of HBCS participants were born during World War II, when Finland became involved in the Winter War (November 1939–March 1940) and the so-called Continuation War (June 1941–September 1944), during which half a million men served in the military forces, that is, 65% of all Finnish men aged 20–49 years [19]. Although air raids caused damage and killed people, Finland was never occupied by the enemy army, unlike many other European countries. However, overcrowding, poor hygienic conditions and a shortage of food made everyday life difficult. Accordingly, mothers and small children were prioritised in food rationing [20,21]. The exceptional circumstances that existed during the war may have evened out socio-economic differences that had previously existed between population groups.

## Methods

The HBCS includes 13,345 people born in Helsinki in either of the two public maternity hospitals: Helsinki University Women’s Hospital or Helsinki City Maternity Hospital. In addition, to be included in the cohort, one had to visit a child welfare clinic in the city and live in Finland in 1971, when a unique identification number was allocated to each resident of the country. The majority of the study participants also attended school in Helsinki. The original cohort has been described elsewhere [22,23]. The study was approved by the Ethics Committee of the National Public Health Institute. Register data were linked with permission from Statistics Finland and the National Institute for Health and Welfare.

### Early-life information

Data obtained from hospital birth records included detailed birth measures and maternal characteristics. We defined the mother’s marital status as married or unmarried at the time of giving birth based on information from birth records as described [7]. We excluded mothers who were recorded as dead (*n*=6), widowed (*n*=22) or divorced (*n*=17) or who had unclear entries (*n*=13). We also excluded 32 married mothers with no information on the male caregiver’s occupation and six unmarried mothers with no information on the mother’s occupation. Our final analyses included 12,612 children born to married mothers and 637 (5.9%) children born out of wedlock. Information on breastfeeding was obtained from child welfare clinic records and dummy coded as follows: not breastfed, breastfed less than three months, breastfed three to six months, breastfed six months or more or no information on breastfeeding available. We also used the mother’s highest occupational status which was obtained from birth, childhood welfare clinic and school records [7]. Occupational status was grouped according to the classification system of Statistics Finland [24] and dummy coded as follows: clerical (employer, self-employed, senior and junior clerical), other (housewife, student, unknown profession) and manual worker, with the latter serving as the reference group [7,25]. As a covariate in our analyses, we used information on 1770 study participants who, as children, were evacuated without their parents to temporary foster homes in Sweden or Denmark during World War II [26].

### Mortality and morbidity

Using personal identification numbers, we followed the subjects through national registers from 1971 to 2014 and identified deaths and hospital admissions from CHD and stroke. We used International Classification of Diseases (ICD) codes 410–414 in ICD-8 and ICD-9 and I21–25 in ICD-10 to define CHD and codes 430–439 in ICD-8 and ICD-9 and I60–69 in ICD-10 to define stroke [27,28]. In Finland, all inpatient hospital admissions are recorded in the Care Register for Health Care. All-cause mortality data were obtained from the National Mortality Register.

### Other adulthood information

Using the personal identification number, we obtained information from Statistics Finland on the subjects’ educational attainment, occupational status, income and marital status at five-year intervals between 1970 and 2000. We used the highest educational attainment, occupational status and personal income level achieved. Participants were split into two groups based on marital status in adulthood: ever married (married, divorced or widowed at any of the time points) and never married (none of these entries at any time points) as described [7,25].

### Statistical analyses

We used Cox proportional hazard models as the primary analytical tool with which to examine the associations between maternal marital status at birth and the occurrence of CHD or stroke. Participants were followed from 1971 until the first of these events: death, migration from Finland, hospitalisation for stroke or CHD or reaching 1 January 2014 alive. First, we calculated hazard ratios (HR) separately for two birth periods: those born before World War II (1934–1939) and those born during World War II (1940–1944). Second, we calculated HR for all people born from 1934 to 1944. Men and women were also analysed separately.

The data were analysed with three Cox models. Each Cox model was stratified for year of birth using the Cox regression strata function, and models including both sexes were also stratified for sex. Model 1 was an unadjusted model, with mother’s marital status at birth being the only predictor. Model 2 included the following early life factors associated with out-of-wedlock birth: length of gestation, birth weight, birth order, duration of breastfeeding, evacuation abroad during World War II and the mother’s age at giving birth and occupational status in childhood. In model 3, we further added the subject’s own educational attainment and marital status as adulthood covariates. In addition, in Supplemental Table SI, we also report the independent effects of covariates that were studied in relation to CHD and stroke outcomes.

We tested potential interactions between sex and mother’s marital status, as well as between period of birth and mother’s marital status in association with cardiovascular outcomes.

As supplementary analyses, we repeated our previously published analyses related to out-of-wedlock birth and offspring’s SEP in adulthood, now by period of birth [7]. In these analyses, we used multinomial logistic regression analysis with two models including similar covariates as in the Cox models. Model 1 included child’s year of birth and sex. Model 2 also included length of gestation, birth weight, birth order, duration of breastfeeding, evacuation abroad during World War II and mother’s age at birth and occupational status in childhood.

## Results

### Maternal and childhood characteristics

Unmarried mothers were on average younger, shorter and had a lower body mass index in late pregnancy than married mothers (Table I). Regardless of marital status, the proportion of middle-class mothers, rather than mothers working in manual professions, increased during the follow-up, as did the duration of breastfeeding. In general, children born out of wedlock were born at an earlier gestational age, were more often firstborn and were shorter and lighter than their peers born to married mothers. These differences were independent of birth period.

**Table I.** Maternal and childhood characteristics of the study participants and their mothers (*N*=13,249) according to mother’s marital status at birth and period of birth.

Child	1934–1939			1940–1944		
	Born to a married mother	Born out of wedlock	*p*–Values	Born to a married mother	Born out of wedlock	*p*–Values
	*M* (*SD*)/*n* (%)	*M* (*SD*)/*n* (%)		*M* (*SD*)/*n* (%)	*M* (*SD*)/*n* (%)	
	*N*=3812	*N*=240		*N*=8800	*N*=397	
Birth weight (g)	3399 (481)	3239 (481)	<0.001	3420 (477)	3296 (456)	<0.001
Head circumference (cm)	35.0 (1.5)	34.7 (1.6)	0.003	35.0 (1.5)	34.7 (1.5)	<0.001
Length at birth (cm)	50.2 (1.9)	49.6 (2.0)	<0.001	50.3 (1.9)	49.9 (1.9)	<0.001
Gestational age (weeks)	39.7 (1.9)	39.3 (2.4)	0.007	40.0 (1.8)	39.7 (2.3)	0.018
Ponderal index	26.8 (2.1)	26.6 (3.0)	0.169	26.8 (2.3)	26.4 (2.3)	0.004
Birth order, first born	1920 (50.4%)	178 (74.2%)	<0.001	4009 (45.6%)	317 (79.8%)	<0.001
Duration of breastfeeding			<0.001			<0.001
Not breastfed	529 (14%)	67 (28%)		1525 (17.5%)	83 (21.1%)	
Less than three months	1315 (34.5%)	78 (32.5%)		2078 (23.6%)	123 (31%)	
Three to six months	864 (22.7%)	43 (17.9%)		2052 (23.3%)	84 (21.1%)	
More than six months	1077 (28.3%)	51 (21.3%)		3059 (34.8%)	104 (26.2%)	
No information available	27 (0.7%)	1 (0.4%)		86 (1.0%)	3 (0.8%)	
Temporary evacuation abroad to a foster family during World War II	982 (26.4%)	54 (23%)		689 (7.9%)	54 (11.5)	
Marital status in adulthood			0.081			0.009
Ever married	3194 (90.8)	184 (87.2)		7355 (89.5)	303 (85.1)	
Never married	323 (9.2)	27 (12.8)		865 (10.5)	53 (14.9)	
Marital status in adulthood			0.595			0.942
Ever divorced	929 (29.1)	65 (30.8)		2493 (33.9)	120 (33.7)	
Never divorced	2265 (70.9)	146 (69.2)		4862 (66.1)	236 (66.3)	
Mother	Married	Unmarried		Married	Unmarried	
		*M* (*SD*)/*n* (%)	*M* (*SD*)/*n* (%)	*M* (*SD*)/*n* (%)	*M* (*SD*)/*n* (%)	
		*N*=3812	*N*=240	*N*=8800	*N*=397	
Height (cm)	159.8 (5.7)	159.0 (6.2)	0.039	160.0 (5.6)	159.1 (6.6)	0.017
Weight (kg)	68.2 (9.1)	65.4 (8.4)	<0.001	66.6 (7.9)	65.6 (8.0)	0.023
BMI (kg/m^2^)	26.7 (3.1)	25.8 (2.7)	<0.001	26.0 (2.8)	25.9 (2.6)	0.408
Age at delivery (years)	27.9 (5.2)	26.1 (5.8)	<0.001	28.8 (5.5)	26.7 (5.7)	<0.001
Mother’s occupation			<0.001			<0.001
Housewives	393 (10.3%)	0		562 (6.4%)	0	
Employers	1 (0%)	0		2 (0%)	0	
Self-employed	31 (0.8%)	2 (0.8%)		74 (0.8%)	4 (1.0%)	
Senior officials	88 (2.3%)	2 (0.8%)		288 (3.3%)	5 (1.3%)	
Junior officials	1204 (31.6%)	43 (17.9%)		3159 (35.9%)	98 (24.7%)	
Manual workers	1973 (51.8%)	193 (80.4%)		4362 (49.6%)	290 (73%)	
Students	33 (0.9%)	0		49 (0.6%)	0	
Missing or unknown	89 (2.3%)	0		304 (3.5%)	0	

*SD*: standard deviation; BMI: body mass index.

### Cardiovascular health in adulthood based on period of birth

We compared offspring’s risk of developing CHD or stroke in later life based on whether they were born before or during World War II. Table II shows HRs and 95% confidence intervals (CIs) for CHD and stroke among children born out of wedlock compared to children born to married mothers according to period of birth and sex. Children born out of wedlock before World War II had a higher risk of developing either CHD or stroke compared to those born to married mothers during the same time period. The interaction between mother’s marital status at birth and period of birth was statistically significant for stroke (*p*=0.015) and for CHD (*p*=0.003).

**Table II.** Number of stroke and CHD events and hazard ratios (with 95% confidence intervals) in people to born out of wedlock as compared with people born to married mothers in total and according to sex and period of birth.

	Stroke			CHD		
	1934–1944	1934–1939	1940–1944	1934–1944	1934–1939	1940–1944
*All*						
Number of people born out of wedlock/total	637/13,249	240/4052	397/9197	637/13,249	240/4052	397/9197
Number (%) of stroke/CHD cases born out of wedlock/total	61 (9.6)/1057 (8.0)	38 (15.8)/417 (10.3)	23 (5.8)/640 (7.0)	83 (13.0)/1588 (12.0)	52 (21.7)/626 (15.4)	31 (7.8)/962 (10.5)
Model 1	1.25 (0.96–1.62)	**1.70 (1.22–2.38)**	0.87 (0.58–1.33)	1.13 (0.90–1.41)	**1.53 (1.15–2.04)**	0.79 (0.55–1.13)
Model 2	1.22 (0.93–1.61)	**1.56 (1.09–2.24)**	0.94 (0.61–1.43)	1.05 (0.83–1.33)	**1.45 (1.07–1.97)**	0.72 (0.49–1.31)
Model 3	1.15 (0.88–1.52)	**1.44 (1.00–2.07)**	0.89 (0.58–1.36)	0.99 (0.78–1.25)	**1.37 (1.02–1.86)**	0.70 (0.48–1.03)
*Men*						
Number of people born out of wedlock/total	326/6921	123/2091	203/4830	326/6921	123/2091	203/4830
Number (%) of stroke/CHD cases born out of wedlock/total	39 (12)/661 (9.6)	24 (19.5)/249 (11.9)	15 (7.4)/412 (8.5)	57 (17.5)/1211 (17.5)	36 (29.3)/458 (21.9)	21 (10.3)/753 (15.6)
Model 1	1.32 (0.96–1.83)	**1.88 (1.23–2.86)**	0.91 (0.54–1.52)	1.03 (0.79–1.35)	**1.49 (1.06–2.10)**	0.68 (0.43–1.04)
Model 2	1.31 (0.93–1.85)	**1.83 (1.15–2.92)**	0.97 (0.57–1.64)	0.94 (0.70–1.25)	1.44 (0.99–2.08)	0.59 (0.37–0.95)
Model 3	1.23 (0.87–1.74)	**1.66 (1.03–2.68)**	0.94 (0.56–1.59)	0.88 (0.66–1.18)	1.33 (0.92–1.93)	0.56 (0.35–0.90)
*Women*						
Number of people born out of wedlock/total	311/6328	117/1961	194/4367	311/6328	117/1961	194/4367
Number (%) of stroke/CHD cases born out of wedlock/total	22 (7.1)/396 (6.3)	14 (12)/168 (8.6)	8 (4.1)/228 (5.2)	26 (8.4)/377 (6.0)	16 (13.7)/168 (8.6)	10 (5.2)/209 (4.8)
Model 1	1.13 (0.74–1.75)	1.47 (0.85–2.54)	0.82 (0.40–1.66)	1.42 (0.95–2.12)	1.62 (0.97–2.73)	1.19 (0.63–2.25)
Model 2	1.13 (0.73–1.76)	1.22 (0.69–2.18)	0.91 (0.45–1.87)	1.37 (0.90–2.06)	1.50 (0.87–2.58)	1.19 (0.62–2.27)
Model 3	1.06 (0.68–1.66)	1.15 (0.64–2.04)	0.86 (0.42–1.76)	1.29 (0.85–1.95)	1.40 (0.81–2.42)	1.13 (0.59–2.16)

Model 1 unadjusted model.

Model 2 adjusted for length of gestation, birth weight, birth order, mother’s age, duration of breastfeeding (dummy coded to no; <3; 3 to 6 and >6 months; no information available), mother’s occupational status in childhood (dummy coded to housewives, students and others; employers, self-employed, senior and junior clericals; workers) and information regarding whether subject was evacuated abroad without parents during World War II.

Model 3 adjusted as in model 2+highest achieved educational status in adulthood and marital status in adulthood (dummy coded to ever married if subject had an entry indicating subject was married, divorced or widowed at any of the time points or to never married if subject had none of these entries at any time points).

CHD: coronary heart disease. Bolded p<0.05.

We performed a secondary analysis separately among men and women. Among men born before World War II, the association was more robust for stroke, while for CHD, the adjustments for childhood SEP attenuated the association to a borderline significant level. Among women, point estimates of HRs were higher for those born before World War II, although they did not reach statistical significance. No associations between out-of-wedlock birth and CHD or stroke in adulthood were seen among those born between 1940 and 1944. We found no interaction between sex and mother’s marital status at birth. However, the interaction between mother’s marital status at birth and period of birth was statistically significant among men for stroke (*p*=0.032) and CHD (*p*=0.005).

### Cardiovascular health in adulthood among people born from 1934 to 1944

We found no association between out-of-wedlock birth and CHD or stroke in adulthood among people born from 1934 to 1944 (Table II). Univariate effects of covariates with CHD and stroke outcomes are presented in Supplemental Table SI.

### SEP in adulthood

As supplementary analyses, we repeated our previously published [7] analyses related to out-of-wedlock birth and SEP in adulthood by period of birth (Tables III and IV). Table IV presents adjusted odds ratios and 95% CIs predicting the highest educational attainment, occupational status and maximum income in thirds in adulthood according to the period of birth and mother’s marital status at birth. These novel analyses confirm that in terms of SEP in adulthood, children born out of wedlock fared worse than children born to married mothers in both periods. However, for educational attainment and occupation but not income, the associations were stronger among those born during the pre-war years. Lower adult SEP was also independently associated with increased risk of both stroke and CHD in later life (Supplemental Table SII).

**Table III.** Number of people born in 1934–1939 and in 1940–1944 according to mother’s marital status at birth and offspring’s educational attainment, occupational status and income in thirds in adulthood.

		Educational attainment in adulthood				
		Upper tertiary	Lower tertiary	Upper secondary	Basic or less or unknown	All subjects
Number of people born out of wedlock/total	1934–1939	6/337	22/788	55/810	128/1793	211/3728
	1940–1944	19/963	65/1938	107/2250	165/3425	356/8576
Number of people born out of wedlock/born to a married mother (%)	1934–1939	2.8%/9.4%	10.4%/21.8%	26.1%/21.5%	60.7%/47.4%	100%/100%
	1940–1944	5.3%/11.5%	18.3%/22.8%	30.1%/26.1%	46.3%/39.7%	100%/100%
		Occupational status in adulthood				
		High official	Low official	Self–employed	Manual worker	All subjects
Number of people born out of wedlock/total	1934–1939	9/510	63/1332	24/350	107/1454	203/3646
	1940–1944	27/1017	109/3136	31/841	171/3407	338/8401
Number of people born out of wedlock/born to a married mother (%)	1934–1939	4.4%/14.6%	31.0%/36.9%	11.8%/9.5%	52.7%/39.1%	100%/100%
	1940–1944	8.0%/12.3%	32.2%/37.5%	9.2%/10.0%	50.6%/40.1%	100%/100%
		Income in adulthood in thirds				
		Highest third		Intermediate third	Lowest third	All subjects
Number of people born out of wedlock/total	1934–1939	53/1245		54/968	104/1496	211/3709
	1940–1944	77/2839		135/3113	142/2590	354/8542
Number of people born out of wedlock/total	1934–1939	25.1%/34.1%		25.6%/26.1%	49.3%/39.8%	100%/100%
	1940–1944	21.8%/33.7%		38.1%/36.4%	40.1%/29.9%	100%/100%

Educational attainment and occupational status indicate maximum position as recorded in the National Census at five-year intervals between 1970 and 2000. Maximum income level is based on state taxation from the same period. For each recorded year, taxable incomes were log transformed and standardised separately for men and women.

**Table IV.** Odds ratios and 95% confidence intervals for adulthood socio-economic position for people born out of wedlock from 1934 to 1939 and from 1940 to 1944 compared to people born to married mothers during the same years.

		Educational attainment in adulthood			
		Upper tertiary	Lower tertiary	Upper secondary	Basic or less or unknown
Model 1	1934–1939	Ref.	1.56 (0.63–3.90)	**3.94 (1.68–9.25)**	**4.01 (1.78–9.25)**
	1940–1944	Ref.	**1.73 (1.03–2.90)**	**2.49 (1.52–4.09)**	**2.53 (1.56–4.01)**
Model 2	1934–1939	Ref.	1.38 (0.54–3.52)	**3.00 (1.25–7.20)**	**3.45 (1.48–8.04)**
	1940–1944	Ref.	1.57 (0.92–2.70**)**	**2.05 (1.22–3.45)**	**2.14 (1.28–5.55)**
		Occupational status in adulthood			
		High official	Low official	Self–employed	Manual worker
Model 1	1934–1939	Ref.	**2.63 (1.29–5.35)**	**3.96 (1.82–8.64)**	**4.42 (2.22–8.79)**
	1940–1944	Ref.	1.28 (0.83–1.97)	1.39 (0.82–2.35)	**1.95 (1.29–2.94)**
Model 2	1934–1939	Ref.	2.09 (0.97–4.47)	**3.66 (1.58–8.45)**	**3.64 (1.73–7.67)**
	1940–1944	Ref.	1.03 (0.66–1.60)	1.37 (0.80–2.33)	1.51 (0.98–2.32)
		Income in adulthood in thirds			
		Highest third	Intermediate third		Lowest third
Model 1	1934–1939	Ref.	1.37 (0.93–2.02)		**1.65 (1.18–2.33)**
	1940–1944	Ref.	**1.63 (1.23–2.17)**		**2.08 (1.57–2.77)**
Model 2	1934–1939	Ref.	1.26 (0.82–1.92)		**1.63 (1.12–2.35)**
	1940–1944	Ref.	**1.12 (1.07–1.16)**		**1.88 (1.39–2.55)**

Educational attainment and occupational status indicate maximum position as recorded in the National Census at five-year intervals between 1970 and 2000. Maximum income level is based on state taxation from the same period. For each recorded year, taxable incomes were log transformed and standardised separately for men and women.

Model 1 adjusted for year of birth and sex.

Model 2 adjusted for year of birth and sex, length of gestation, birth weight, birth order, mother’s age, duration of breastfeeding (dummy coded to no; <3; 3 to 6 and >6 months; no information available), mother’s occupational status in childhood (dummy coded to housewives, students and others; employers, self-employed, senior and junior clericals; workers) and information regarding whether the subject was evacuated abroad without parents during World War II.Bolded p<0.05.

## Discussion

Our study shows that people born out of wedlock in a peacetime society in which marriage was the parental norm seem to have a lifelong health disadvantage, as illustrated by higher rates of CHD and stroke in adulthood. This association is only partly explained by other childhood socio-economic indicators or by adult SEP or marital status. However, this relative disadvantage was no longer observed during World War II when society faced a severe strain and exceptional circumstances, such as shortage and rationing. These changes seem to have evened out inequalities that existed in peacetime society.

We also repeated our previous analyses related to adult SEP among those born out of wedlock based on period of birth. In terms of adulthood education and occupation but not income, the disadvantage of being born to an unmarried mother was again largely limited to those born before World War II. The most disadvantaged group consisted of children born out of wedlock during the pre-war period.

Previous studies suggest that a mother’s marital status may involve a different dimension of social stratification regarding her offspring than SEP based on parental education or occupation. According to these studies, out-of-wedlock origin contributes to later life health disparities [7,8–12,14] and may increase the risk of CVD [8,11]. However, methodological differences prevent precise comparisons between these studies. A study from Sweden consisted of men who were born from 1915 to 1929. According to this study, out-of-wedlock birth was associated with higher ischaemic heart disease mortality in adulthood among men who remained unmarried themselves. However, compared to our cohort, these men were less affected by the war because they were born in a stable society that remained neutral in World War II [8]. Another study by Elo et al. showed that living in a mother-only family in 1950 was associated with higher CVD mortality among men and women born in Finland from 1936 to 1950. However, in that study, period of birth also predicted mortality: CVD mortality was higher for people born before the war (HR=1.47) and during the war (HR=1.19) than after the war, when family type was taken into account [11].

A novel finding of our study is that the increased risk of CHD or stroke was found only among those who were born out of wedlock during the pre-war period. The interaction between out-of-wedlock birth and period of birth was statistically significant both for stroke and CHD, implying that the difference between birth periods is very unlikely to have arisen by chance.

It is obvious that the war had a broad impact on society. The disordered conditions and temporary separation precluded, temporarily or permanently, some marriages that would have normally followed a conception. Public attitude towards unmarried mothers may have become more permissive [29]. Narrowing income inequalities, shortages and rationing may have evened out socio-economic differences that existed between population groups before the war. Moreover, evacuation to the countryside to a safer environment and better nutritional care, women’s increased contribution to the labour market and maternity grants most likely benefitted mothers from lower social classes. For these reasons, an out-of-wedlock birth during wartime did not represent the same kind of disadvantage as it did in pre-war society.

This study has several strengths. We measured early life characteristics such as mother’s marital status, if available, from reliable records. When measuring mother’s occupation, we used the highest occupational status during childhood instead of a single measurement from one specific time point. The register data that we used have been validated and found to be accurate for epidemiological studies. However, some limitations to the present study must also be acknowledged. Data on cohabitations without marriage, other family members or children’s place of residence during the war were not available. Occupational data were classified based on modern standards and do not differentiate between skilled and unskilled workers. These data limitations could be expected to increase random error and result in a more conservative estimate. Moreover, the proportion of out-of-wedlock children in our study was lower than in Helsinki during the time period in question. This would again be expected to lead to a more conservative estimate, that is, a more favourable outcome for those born out of wedlock. Due to high infant mortality among children born out of wedlock, many died before our follow-up. This could in theory diminish the difference between those born out of wedlock and those born to a married mother during World War II, but we believe it is unlikely to cause a large enough bias to explain our results.

Finally, even though no direct analogy can be drawn between contemporary and early society in Helsinki, a large number of the people at a higher risk of CVD currently, much like our study participants, are those who are born into societies in which marriage remains the parental norm. Furthermore, from a public health perspective, modern societies may contain minority groups of children experiencing social and economic hardship or stigma with lifelong health effects, similar to those of children born out of wedlock 80 years ago.

## Conclusions

Our study shows an association between birth out of wedlock and CVD in adulthood among people born before World War II. These results indicate that in a society in which marriage is the parental norm, the mother’s marital status may be an important indicator of early life disadvantage, and that this may vary rapidly when societal circumstances change. Together with other measures, such as parental occupational status, it reflects unfavourable early life circumstances from an alternative viewpoint. The disadvantage that children of unmarried mothers experienced 80 years ago is likely to persist throughout their entire lives.

## Supplemental Material

sj-docx-1-sjp-10.1177_14034948211019792 – Supplemental material for Cardiovascular disease in people born to unmarried mothers in two historical periods: The Helsinki Birth Cohort Study 1934–1944 Click here for additional data file. Supplemental material, sj-docx-1-sjp-10.1177_14034948211019792 for Cardiovascular disease in people born to unmarried mothers in two historical periods: The Helsinki Birth Cohort Study 1934–1944 by H. Maiju Mikkonen, Minna K. Salonen, Antti Häkkinen, Clive Osmond, Johan G. Eriksson and Eero Kajantie in Scandinavian Journal of Public Health

sj-docx-2-sjp-10.1177_14034948211019792 – Supplemental material for Cardiovascular disease in people born to unmarried mothers in two historical periods: The Helsinki Birth Cohort Study 1934–1944 Click here for additional data file. Supplemental material, sj-docx-2-sjp-10.1177_14034948211019792 for Cardiovascular disease in people born to unmarried mothers in two historical periods: The Helsinki Birth Cohort Study 1934–1944 by H. Maiju Mikkonen, Minna K. Salonen, Antti Häkkinen, Clive Osmond, Johan G. Eriksson and Eero Kajantie in Scandinavian Journal of Public Health
