# Supplementary material for: Cardiovascular disease in people born to unmarried mothers in two historical periods: The Helsinki Birth Cohort Study 1934–1944
Source: Scand J Public Health. 2021 May 31;50(5):613–21. doi: 10.1177/14034948211019792 (PMC9203658; doi:10.1177/14034948211019792)
Supplement: sj-docx-1-sjp-10.1177_14034948211019792 – Supplemental material for Cardiovascular disease in people born to unmarried mothers in two historical periods: The Helsinki Birth Cohort Study 1934–1944 [file sj-docx-1-sjp-10.1177_14034948211019792.docx]

| Supplementary Table SI. Hazard ratios and 95% confidence intervals of covariates with stroke and coronary heart disease outcomes. | | |
| --- | --- | --- |
|  | **Stroke** | **Coronary heart disease** |
| Sex (female vs. male) | .59 (.52–.67) | .30 (.27–.34) |
| Year of birth | .99 (.97–1.02) | .96 (.95–.98) |
| Birth order (later vs. firstborn) | 1.02 (.91–1.16) | 1.04 (.94–1.15) |
| Birth weight (kg) | .80 (.71–.91) | .83 (.75–.92) |
| Mother’s age (years) | 1.00 (.98–1.01) | .99 (.98–1.00) |
| Length of gestation (weeks) | .99 (.95–1.02) | 1.00 (.97–1.03) |
| Duration of breastfeeding |  |  |
| Not breastfed | Ref. | Ref. |
| Less than three months | .99 (.82–1.20) | .94 (.80–1.10) |
| Three to six months | .94 (.76–1.15) | .92 (.78–1.09) |
| More than six months | 1.01 (.83–1.22) | .96 (.83–1.12) |
| No information available | 1.10 (.58–2.01) | .78 (.43–1.43) |
| Mother’s occupation |  |  |
| Manual worker | Ref. | Ref. |
| Housewives, students and others | .80 (.65–.99) | .83 (.70–.98) |
| Employers, self–employed, senior and junior clericals | .85 (.74–.97) | .81 (.73–.91) |
| Information about whether subject was evacuated abroad during World War II (yes vs. no) | 1.03 (.86–1.23) | 1.07 (.92–1.23) |
| Educational attainment in adulthood |  |  |
| Basic or less or unknown | Ref. | Ref. |
| Upper secondary | .92 (.79–1.06) | .81 (.72–.93) |
| Lower tertiary | .71 (.60–.84) | .65 (.56–.74) |
| Upper tertiary | .59 (.46–.74) | .42 (.34–.51) |
| Own marital status in adulthood (ever married vs. never married) | .89 (.72–1.10) | .82 (.70–.97) |
