# Supplementary material for: Cardiovascular disease in people born to unmarried mothers in two historical periods: The Helsinki Birth Cohort Study 1934–1944
Source: Scand J Public Health. 2021 May 31;50(5):613–21. doi: 10.1177/14034948211019792 (PMC9203658; doi:10.1177/14034948211019792)
Supplement: sj-docx-2-sjp-10.1177_14034948211019792 – Supplemental material for Cardiovascular disease in people born to unmarried mothers in two historical periods: The Helsinki Birth Cohort Study 1934–1944 [file sj-docx-2-sjp-10.1177_14034948211019792.docx]

| Supplementary Table SII. Hazard ratios and 95% confidence intervals of adulthood education, occupation and income for stroke and coronary heart disease. | | |
| --- | --- | --- |
|  | STROKE | CHD |
| Educational attainment in adulthood | | |
| Basic or less or unknown | Ref. | Ref. |
| Upper secondary | .92 (.79–1.06) | .81 (.72–.93) |
| Lower tertiary | .71 (.60–.84) | .65 (.56–.74) |
| Upper tertiary | .59 (.46–.74) | .42 (.34–.51) |
| Income in adulthood in thirds | | |
| Lowest third | Ref. | Ref. |
| Intermediate third | .68 (.58–.78) | .59 (.55–.70) |
| Highest third | .59 (.50–.68) | .55 (.49–.63) |
| Occupation in adulthood | | |
| Manual worker | Ref. | Ref. |
| Self-employed | .76 (.61–.94) | .78 (.66–.93) |
| Low official | .73 (.63–.84) | .71 (.63–.80) |
| High official | .54 (.44–.68) | .52 (.44–.62) |
